# Long-Term Resveratrol Supplementation as a Secondary Prophylaxis for Stroke

**DOI:** 10.1155/2018/4147320

**Published:** 2018-03-18

**Authors:** Katalin Fodor, Delia Mirela Tit, Bianca Pasca, Cristiana Bustea, Diana Uivarosan, Laura Endres, Ciprian Iovan, Mohamed M. Abdel-Daim, Simona Bungau

**Affiliations:** ^1^Pharmacy Department, Faculty of Medicine and Pharmacy, University of Oradea, 410028 Oradea, Romania; ^2^Preclinical Disciplines Department, Faculty of Medicine and Pharmacy, University of Oradea, 410028 Oradea, Romania; ^3^Pharmacology Department, Faculty of Veterinary Medicine, Suez Canal University, Ismailia 41522, Egypt; ^4^Department of Ophthalmology and Micro-technology, Yokohama City University, Yokohama, Japan

## Abstract

Stroke is a leading cause of mortality worldwide, as well as a source of long-term disabilities and huge socioeconomic costs. This study investigates the effects of resveratrol, an antioxidant supplement, on blood pressure, weight status, glucose, and lipid profile in patients who had a stroke in the last 12 months. Two hundred and twenty-eight patients were divided into three groups: group I received only allopathic treatment (control group), while groups II and III received allopathic treatment with a daily supplementation of oral resveratrol (100 and 200 mg, resp.) for 12 months. In all groups, the changes of the studied parameters were monitored at 6 and 12 months from the initial evaluation. In groups II and III, resveratrol induced significant changes (*p* < 0.05) in the blood pressure, body mass index, as well as all parameters of the lipid profile, and glucose (in nondiabetic patients), compared to the control group. The supplementation of the allopathic treatment with resveratrol had a beneficial effect on all monitored parameters, which serve as major risk factors for stroke.

## 1. Introduction

Neurodegenerative, circulatory, and cardiovascular diseases and cancers are considered as direct consequence of the complex of phenomena, named “oxidative stress” [1–3]. Among these, stroke represents a leading cause of mortality worldwide and a major source of long-term disabilities and huge socioeconomic costs [4]. A recent study has revealed that about 90% of strokes can be attributed to the presence of 10 risk factors, including high blood pressure (BP), dyslipidemias, consumption of toxic substances (alcohol and tobacco), obesity, daily stress, sedentariness, and diabetes mellitus [5].

During the pathophysiology of stroke, reactive oxygen species (ROS) are generated, which can trigger chain reactions that destroy the neuronal membranes [6, 7]. There are cumulative evidences suggesting that ROS can damage the cellular components [8], enhance the production of inflammatory mediators which in turn can lead to additional oxidative stress [9–12], and are involved in all the pathophysiological stages of neuronal death [13]. During ischemia, mitochondria (the main ROS-generating cellular components) suffer dysfunction, which causes an increase in oxidative stress. Increased production of ROS through mitochondria plays a role in the pathogenesis of stroke through direct damage to biomolecules resulting in necrosis, necroptosis and apoptosis, damaged endothelium-dependent vasodilator mechanisms, induction of mitochondrial permeability of transition, and interrupted excitation-contraction coupling [14].

Reactive oxygen species have an important role in normal physiological processes, being also implicated in a lot of disease processes, where they mediate damage to cell structures, including membranes, lipids, deoxyribonucleic acid (DNA), and proteins. Oxidative stress has an important role in the pathogenesis of ischemic brain injury that follows a cerebrovascular attack, having as goal the cerebral vasculature. The primary reactive oxygen species (like superoxide) and its derivatives, in animal models with ischemic stroke, cause vasodilatation by opening the potassium channels, altering the vascular reactivity, and breaking down the blood-brain barrier [15].

Diabetes mellitus and atherosclerosis are diseases that are associated with chronic inflammation produced by ROS [11, 16]. Consequently, stroke therapies should also consider secondary prophylaxis by addressing ROS on the medium and long term.

To neutralize these free radicals, the presence of potentially neutralizing agents (antioxidants) in the body is needed [17]. Particular attention is paid to the use of natural antioxidant agents that can be administered safely in humans in determined, verified, and standardized doses [18, 19]. Resveratrol (3,5,4′-trihydroxy-trans-diphenyl-ethylene) is well-known as a natural antioxidant, effective in combating oxidative stress and related inflammation [20, 21]. Due to its antioxidant effect, this substance can neutralize free radicals in the human body, thus reducing the resultant cellular aging process [22–24]. Resveratrol possesses phenolic functions (Ar-OH) susceptible to block-connect hydroperoxide radicals resulting from lipid peroxidation [25].

Numerous studies have shown the effectiveness of resveratrol in improving health and preventing chronic diseases. However, it is unclear whether these effects persist with prolonged administration of resveratrol [20, 26]. This study investigates the effects of long-term resveratrol supplementation on BP, weight status, glucose, and lipid profile in patients who had a stroke in the last 12 months.

## 2. Methods

### 2.1. Study Design

This study included patients who had first stroke in the last 12 months, hospitalized for recovery treatment during 2011–2015 (patients were recruited each year during 5 years, each patient being under observation for a year). All patients were clinically stabilized after stroke. When they were included in this study, the patients were hospitalized in the *Baile Felix Clinical Rehabilitation Hospital* where they followed a complex medical physical rehabilitation program that imposed restrictions for BP values.

The research was conducted in accordance with the WMA Ethical Declaration of Helsinki and was approved by the Ethics Commission of the Council of Medicine and Pharmacy Faculty, University of Oradea, Romania. Each patient included in this study signed an informed consent form, before the inclusion.

Two hundred and twenty-eight patients were divided into three groups: *group I* (control), (*n* = 92 patients) who underwent allopathic (medical) treatment combined with medical physical rehabilitation, while *groups II* (*n* = 81 patients) and *III* (*n* = 55 patients) received allopathic treatment and medical physical rehabilitation, combined with a 12-month supplementation with an oral daily dose of resveratrol (100 and 200 mg/patient, resp.). The patients were distributed into groups using the method of selection for each group, depending on the patient's willingness of taking 100 or 200 mg resveratrol, according to every patient's history and clinical characteristics, so that there were no significant differences between groups. Allopathic treatment was individualized for each patient, depending on the clinical characteristics and associated pathology. All patients were evaluated initially, at 6 months and at 12 months from the beginning of the treatment. The following parameters were monitored: BP, weight status, lipid profile (total cholesterol, high-density lipoprotein (HDL) cholesterol, low-density lipoprotein (LDL) cholesterol, and triglycerides), and glucose profile.

### 2.2. Clinical Investigations

#### 2.2.1. Blood Pressure Measurement

Blood pressure measurement was performed following the 2007 Guidelines for the Management of Arterial Hypertension [27].

#### 2.2.2. Body Mass Index (BMI) Determination

To determine the patients' weight status, the BMI was calculated using the following equation (BMI = weight in kilograms divided by the square of height in meters, expressed in kg/m^2^) [28].

### 2.3. Biochemical Analysis

Blood samples were obtained in the morning after a 12–14-hour meal break and were used to evaluate the levels of total cholesterol, triglycerides, HDL and LDL cholesterol, basal glucose, and glycosylated hemoglobin (HbA1c).

#### 2.3.1. Lipid Profile

The cholesterol, triglycerides, HDL cholesterol, and LDL cholesterol assays were performed on the Beckman Coulter AU680 analyser using Beckman Coulter reagents, produced by Beckman Coulter Inc., Ireland. The methods of determination included the oxidase-peroxidase method for cholesterol, enzymatic glycerol-3-phosphate oxidase method for triglycerides, and colorimetric direct method for HDL and LDL cholesterol.

#### 2.3.2. Fasting Blood Glucose

Basal glucose was determined by the hexokinase method, the values on an empty stomach being used for the selection of all subjects. A normal level of basal blood glucose was considered a plasmatic concentration of <100 mg/dL [29].

#### 2.3.3. Glycosylated Hemoglobin

Glycosylated hemoglobin was determined only for diabetic patients. It is a real image of glucose control over a period of 90–120 days retrospectively [26]. Determination was performed from venous blood collected on EDTA anticoagulant by HPLC (Gold Standard) using BioRad D-10 equipment and reagents.

#### 2.3.4. Quality Control

The internal quality control was performed according to ISO 15189: 2013 standard. Variation coefficients were as follows: cholesterol CV% = 2.05, triglyceride CV% = 1.95, HDL cholesterol CV% = 2.29, LDL cholesterol CV% = 1.74, glucose CV% = 2.05, and HbA1c CV% = 0.82.

### 2.4. Statistical Analysis

Statistical analysis was done using EPIINFO, version 6.0, a program of the Center for Disease Control and Prevention from Atlanta, adapted to the medical statistics processing. Average parameter values, frequency ranges, standard deviations, and statistical significance tests by the Student method (*t* test) and χ^2^ were calculated.

The distribution of the tests is similar to normal, being used by assumptions involving numerical data. The *t* test used was paired *t* test. In order to obtain an indicator independent of the measurement units of the two variables, the Bravais-Pearson correlation coefficient was used. Statistical significance was assigned at a *p* value of <0.05.

In order to interpret the magnitude of change in parameters at different time points, the “effect size” (ES) was measured. Based on statistical literature, the interpretation of this index was compiled, namely, small ES = 0.20, medium ES = 0.50, and large ES = 0.80. When expressing the findings of a quantitative study, ES is important because a *p* value can identify if there is an effect but cannot reveal its magnitude [30, 31].

## 3. Results

### 3.1. Patients' Characteristics

In terms of demographic and clinical characteristics, there were no significant differences (*p* > 0.05) between the three groups (Table 1**)**.

### 3.2. Clinical Evaluations

#### 3.2.1. The Effect of Resveratrol on BP

Initially, there were no significant differences (*p* > 0.05) in the mean BP values between the three experimental groups (Table 2).

*(1) Systolic BP*. In the group receiving 100 mg resveratrol, the systolic BP value decreased from 148.02 mmHg to 143.12 mmHg at 6 months (*p* < 0.05) and to 139.85 mmHg in the next 6 months (*p* < 0.05). In the group receiving 200 mg resveratrol, the systolic BP value decreased from 149.21 mmHg to 142.02 mmHg at 6 months (*p* < 0.05) and to 139.35 mmHg in the next 6 months (*p* < 0.05). In the control group, systolic BP values decreased from 148.42 mmHg to 146.10 mmHg at 6 months (*p* > 0.05) and to 145.32 mmHg in the next 6 months (*p* > 0.05).

Comparing data (Table 2) at the initial evaluation and at 12 months of treatment, the ES was 0.53 in the group receiving 100 mg resveratrol, ES = 0.53 (*p* < 0.05) and 0.65 in the group receiving 200 mg resveratrol (*p* < 0.05), while in the control group, ES was 0.20 (*p* > 0.05).

*(2) Diastolic BP*. In the group receiving 100 mg resveratrol, the diastolic BP value decreased from 88.29 mmHg to 85.89 mmHg at 6 months (*p* > 0.05) and to 84.27 mmHg in the next 6 months (*p* > 0.05). In the group receiving 200 mg resveratrol, the diastolic BP value decreased from 88.47 mmHg to 85.91 mmHg at 6 months (*p* > 0.05) and 84.10 mmHg in the next 6 months (*p* > 0.05). In the control group, the diastolic BP value decreased from 87.61 mmHg to 86.39 mmHg at 6 months (*p* > 0.05) and 85.67 mmHg in the next 6 months (*p* > 0.05), respectively (Table 2).

Comparing data at the initial evaluation and at 12 months of treatment, ES was 0.37 in the group with 100 mg resveratrol (*p* < 0.05), 0.39 in the group receiving 200 mg resveratrol (*p* < 0.05), and 0.17 for the control group without resveratrol supplementation (*p* > 0.05).

#### 3.2.2. The Effect of Resveratrol on the Weight Status and BMI

Initially, there were no significant differences (*p* > 0.05) in BMI values between the two experimental groups. After a 12-month administration of resveratrol at 100 and 200 mg, we recorded a significant decrease (*p* < 0.05) in the mean BMI values, compared to the control group (Table 3).

The mean BMI values decreased after 6 months in the group receiving 100 mg resveratrol from 29.47 kg/m^2^ to 27.97 kg/m^2^ (*p* > 0.05), resulting in an ES = 0.35; in the group receiving 200 mg resveratrol, from 29.50 kg/m^2^ to 27.56 kg/m^2^ (*p* > 0.05), resulting in an ES = 0.45; in the nonresveratrol group, the BMI had a minimal decrease from 29.95 kg/m^2^ to 29.42 kg/m^2^ (*p* > 0.05), resulting in an ES = 0.12.

In the next 6 months, in the group receiving 100 mg resveratrol, a decrease in BMI was noticed from 27.97 kg/m^2^ to 26.75 kg/m^2^ (*p* > 0.05), resulting in an ES = 0.27; in the group receiving 200 mg resveratrol, a decrease in BMI was noticed from 27.56 kg/m^2^ to 25.77 kg/m^2^ (*p* < 0.05), resulting in an ES = 0.42; in the group without resveratrol, a decrease in BMI was noticed from 29.42 kg/m^2^ to 29.31 kg/m^2^ (*p* > 0.05), resulting in an ES = 0.02.

Comparing data at the initial evaluation and at 12 months of treatment, ES was 0.64 (from 29.47 kg/m^2^ to 26.75 kg/m^2^, *p* < 0.05) in the group receiving 100 mg resveratrol, 0.87 (from 29.50 kg/m^2^ to 25.77 kg/m^2^, *p* < 0.05) in the group receiving 200 mg resveratrol, and 0.14 (from 29.95 kg/m^2^ to 29.31 kg/m^2^, *p* > 0.05) in the group without resveratrol supplementation (Table 3).

### 3.3. Biochemical Analysis

#### 3.3.1. The Effect of Resveratrol on the Lipid Profile

In all groups, the lipid profile improved significantly both at 6 months and at 12 months (*p* < 0.05). In the resveratrol groups, the lipid profile improved significantly over the 12 months (*p* < 0.05), compared with the control group (Table 4). After 12 months, resveratrol administration irrespective of the dose had a substantial effect on all lipid profile parameters, especially on triglycerides (ES = 1.81 at 100 mg and ES = 1.95 at 200 mg) and serum cholesterol (ES = 1.51 at 100 mg and ES = 1.63 at 200 mg). For the control group, the ES was greater than 1 as well, in the case of triglycerides and serum cholesterol (ES = 1.39 and ES = 1.07, resp.). Compared to the control group, the effect of resveratrol 100 mg was 2.02 times greater for HDL cholesterol (ES = 1.25 versus ES = 0.62) and of 2.34 for LDL cholesterol (ES = 1.24 versus ES = 0.53). Also, compared to the control group, the effect of resveratrol 200 mg was 2.11 times greater for HDL-cholesterol (ES = 1.31 versus ES = 0.62) and of 2.42 times for LDL-cholesterol (ES = 1.28 versus ES = 0.53).

#### 3.3.2. The Effect of Resveratrol on the Glycemic Profile

*(1) Basal Glycemia*. In diabetic patients, blood glucose levels decreased insignificantly (*p* > 0.05) after 6 months, in the group receiving 100 mg resveratrol (from 142.18 mg/dL to 136.29 mg/dL, ES = 0.39) and in the group receiving 200 mg (from 142.46 mg/dL to 136.21 mg/dL, ES = 0.41). The decrease was significant compared to the control group where the blood glucose values had a minimal decrease (from 142.47 mg/dL to 141.81 mg/dL, ES = 0.04).

In the following 6 months, blood glucose decreased in the group receiving 100 mg resveratrol from 136.29 to 134.24 mg/dL, ES = 0.17, and in the group receiving 200 mg from 136.21 to 133.89 mg/dL, ES = 0.16. In the group without resveratrol, the effect was ES = 0.08 (from 141.81 to 140.58 mg/dL).

Comparing the results of the initial evaluation and 12 months of assessment in diabetic patients, ES was 0.52 (from 142.18 mg/dL to 134.24 mg/dL, *p* > 0.05) in the group receiving 100 mg resveratrol, 0.56 in the group receiving 200 mg resveratrol (from 142.46 mg/dL to 133.89 mg/dL, *p* > 0.05) and 0.12 (from 142.47 mg/dL to 140.58 mg/dL, *p* > 0.05) in the group without resveratrol supplementation.

In nondiabetic patients, blood glucose levels decreased significantly (*p* < 0.05) after 6 months in the group receiving 100 mg of resveratrol (from 96.67 mg/dL to 91.23 mg/dL, ES = 0.48) and in the group receiving 200 mg (from 96.98 mg/dL to 91.21 mg/dL, ES = 0.51). The decrease was significant from the baseline and from the control group where the blood glucose values had a minimal decrease (from 95.71 mg/dL to 94.55 mg/dL, ES = 0.1).

In the following 6 months, blood glucose decreased in the group receiving 100 mg resveratrol from 91.23 to 87.12 mg/dL, ES = 0.38, in the group receiving 200 mg from 91.21 to 86.11 mg/dL, ES = 0.48, and in the group without resveratrol, ES = 0.11 (from 94.55 to 93.33 mg/dL). In both groups with resveratrol, the decrease was significant compared to the value at six months and compared to the control group (Table 5).

Comparing the results of the initial evaluation and 12 months of assessment in nondiabetic patients, ES was 0.85 (from 96.67 mg/dL to 87.12 mg/dL, *p* < 0.05) in the group receiving 100 mg resveratrol, 0.97 (from 96.98 mg/dL to 86.11 mg/dL, *p* < 0.05) in the group receiving 200 mg resveratrol, and 0.20 (from 95.71 mg/dL to 93.33 mg/dL, *p* > 0.05) in the group without resveratrol supplementation.

*(2) Glycosylated Hemoglobin*. Glycated hemoglobin values decreased after 6 months as follows: in the group receiving 100 mg resveratrol from 7.13% to 6.72% (*p* > 0.05, ES = 0.27); in the group receiving 200 mg resveratrol from 7.15% to 6.66% (*p* > 0.05, ES = 0.33); and in the group without resveratrol supplementation, there was a minimal decrease in HbA1c values (from 7.10% to 7.09%, *p* > 0.05, ES = 0.01).

In the next 6 months, a decrease in HbA1c mean values was noticed from 6.72% to 6.61% (*p* > 0.05, ES = 0.08) and from 6.66% to 6.55% (*p* > 0.05, ES = 0.08) in the group receiving 200 mg resveratrol. In the group without resveratrol supplementation, ES was 0.03 (from 7.09% to 7.05%, *p* > 0.05) (Table 6).

Comparing data at the initial evaluation and at 12 months of treatment, ES was 0.34 (from 7.13% to 6.61%, *p* > 0.05) in the group receiving 100 mg resveratrol, 0.40 (from 7.15% to 6.55%, *p* > 0.05) in the group receiving 200 mg resveratrol, and 0.03 (from 7.10% to 7.05%, *p* > 0.05) in the group without resveratrol supplementation.

## 4. Discussion

One of the most prevalent cardiovascular illness is stroke. This is a main problem not only in matter of health, both in terms of sequelae and the destructive impact on the quality of life of the patient, implicitly on his or her relatives, but also in the point of view of increasing mortality.

Comprehensive research over the last few decades has discovered multiple mechanisms by which resveratrol changes cardiovascular risk factors [32, 33]. Diabetes, hypercholesterolemia, and hypertension, by decreasing bioactive nitric oxide, lead to high production of reactive oxygen species in vessel walls. Endothelial dysfunction is a significant mechanism of cerebrovascular injury, deriving in part from the excessive generation of reactive oxygen species [32].

Here, we show that long-term resveratrol supplementation in patients poststroke had a beneficial effect on these parameters that serve as major risk factors for stroke.

To evaluate the long-term dose-dependent resveratrol potential in the secondary prevention of stroke, in this study, we compared the effects of two different doses of resveratrol in terms of effectiveness on BP, weight status, lipid profile, and glucose profile, in patients who have suffered a stroke in the past 12 months.

We chose doses of 100 mg and 200 mg resveratrol/day, respectively, due to the fact that it has been shown that doses of resveratrol lower than 0.5 g per person may be sufficient to decrease blood glucose levels, improve insulin action, and generate cardioprotective effect and other favorable effects [34, 35]. Moreover, some studies imply that in certain cases, resveratrol can be more efficient in lower doses compared to the effect produced by this compound in higher doses [36, 37]. Nevertheless, studies upon the toxicity of resveratrol in humans reveal that doses up to 0.5 g per day for long periods may determine only reversible and moderate side effects, being well tolerated [38].

We recorded significant decreases in BP, BMI, lipid profile parameters, and glucose in patients, treated with a daily oral dose of 100 or 200 mg resveratrol for a year, as compared to the control group. The effect of resveratrol in 200 mg dose was significantly better than in 100 mg dose, on triglyceride values alone. Patients were monitored for a period of 12 months, during which no new vascular attack occurred in any group; however, in the resveratrol groups, the monitored parameters were significantly improved. Previous studies have revealed that stroke risk factor identification and treatment can considerably prevent long-term morbidity and mortality and can diminish ischemic stroke after a first stroke [38, 39].

Besides age, the most significant cardiovascular risk factor for developing both hemorrhagic and ischemic stroke is hypertension. In addition, hypertension predisposes to risk of cardiac diseases and atherosclerosis, thereby determining cerebral embolism. The inflammatory mechanisms hold an important role in the progression and pathogenesis of atherosclerosis, thrombosis, plaque rupture, and stroke [40]. As we have previously mentioned, endothelial dysfunction is regarded as an important mechanism for cerebrovascular damages [32].

Several studies have reported an increased level of lipid peroxidation and oxidative stress biomarkers in patients with essential or secondary hypertension [41–44]. Other studies showed that the production of H_2_O_2_ and 13-hydroxyoctadecadienoic acid, a ROS production marker, is significantly higher in these patients than in subjects with normal BP [45, 46]. Therefore, due to its antioxidant activity, resveratrol may have beneficial effects in reducing high BP. In a study performed on 97 patients, Theodotou et al. concluded that adding resveratrol to the standard antihypertensive treatment effectively reduces BP [47]. This may occur through increasing the production of nitric oxide in blood vessels, where it activates the guanylate cyclase enzyme, which facilitates vasodilatation [40, 48].

Our study demonstrated that the association of resveratrol to allopathic treatment for 12 months resulted in a significant decrease in BP (*p* < 0.05) in poststroke patients.

The evaluations at 6 months and 12 months did not indicate significant differences between the two batches with different doses of resveratrol on the effect on BP.

Multiple studies have demonstrated that resveratrol is able to determine cell cycle arrest and counteracts adipogenesis, in the same time having a proapoptotic effect in adipocytes of mice and humans [49].

Resveratrol is a substance with multiple desirable antiobesity effects on adipocytes in body storage tissues. It also diminishes adipogenesis and viability in preadipocyte maturation, which is mediated by the regulation of specific adipocyte and enzyme-specific transcription factors as well as genes that modulate mitochondrial function. Resveratrol also increases the processes of lipolysis and reduces lipogenesis in mature adipocytes [50].

In this study, following a 12-month administration of resveratrol 100 mg and 200 mg, respectively, there was a significant decrease in mean BMI when compared to the control group, with no significant differences between the groups with 100 and 200 mg. BMI values have changed over the 12 months even though most patients kept their weight, the results showing a decrease of this index.

Dyslipidemia and steatohepatitis determined by the atherogenic diet in mice were improved by resveratrol, its beneficial effects being correlated with the altered expression of hepatic genes implicated in lipid metabolism [51]. In obese mice, given a high-calorie diet, resveratrol significantly decreased the body weight [52–54]. Studies on the nonhuman primate (*Microcebus murinus)* found that the administration of 200 mg/kg/day of resveratrol caused an increase in basal metabolism and energy consumption, suggesting a potential mechanism for weight loss [55, 56]. In this paper, favorable changes in the lipid profile in all three groups, both at 6 months and at 12 months, may be associated with the hygienic-dietary regimen imposed on poststroke patients.

Six months after starting resveratrol administration, a significant decrease in cholesterol and lipid profile change in favor of the patient was observed. Significant values in lipid profile changes were even more noticeable 12 months after resveratrol administration started. In order to define the lipid profile, a great importance had the increase in HDL cholesterol values and decrease in LDL cholesterol, respectively, after the administration of resveratrol. Triglyceride levels decreased significantly in the groups of patients with resveratrol. Between the two resveratrol groups, there were significant differences in triglyceride values, which meaningfully decreased in the group with 200 mg/day/patient.

In the literature, it has been highlighted that resveratrol has antidiabetic effects, with a positive effect on both insulin and pancreatic *β* cells, and impedes complications of the disease [57, 58]. Resveratrol activates the Sirtuin 1 (SIRT1) pathways, according to some recent studies on how resveratrol works in diabetes. SIRT1 expression and activity were significantly reduced in experimental models of diabetes mellitus. Some of the favorable effects of resveratrol on adjusting glucose homeostasis are mediated by the activation of adenosine monophosphate-activated kinase (AMPK). Modification of AMPK activity, under hyperglycemic conditions, correlates with insulin resistance and tissue damage and with hyperglycemia, supporting a key role of AMPK in type 2 diabetes [59].

Data existing in the literature show diverse effective doses of resveratrol in insulin-resistant individuals. Thus, it is important to understand that the mechanisms of action of resveratrol can vary according to its dose. Treatment with lower doses of resveratrol activates SIRT1, while higher doses activate AMPK in a way independent of SIRT1 [60].

We noticed a decrease in blood glucose levels, both in the first and last six months of administration. The changes were not significant in diabetic patients for none of the studied groups; however, the effect was more favorable in the resveratrol groups. Oxidative stress plays an important role in the development of insulin resistance and can be neutralized by the antioxidant effect of resveratrol [59, 61]. Previous studies indicated increased insulin sensitivity in patients treated with resveratrol [62] and explained these effects by the decrease in oxidative stress, as evidenced by the monitoring of some biochemical parameters.

**Figure 1:**
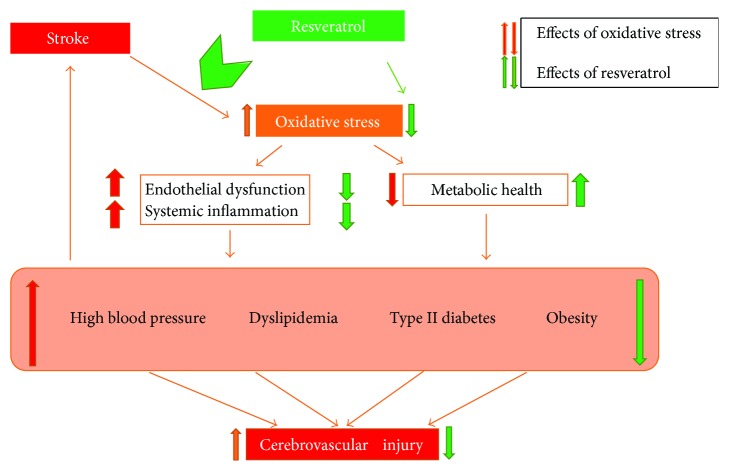


Recommendations: six months after starting resveratrol administration, a significant decrease in cholesterol and LDL cholesterol was observed. Thus, adding resveratrol to standard lipid-lowering medication may help restoring normal lipid profile values in these patients (Figure 1). Observation of the lifestyle and nutrition subsequent to a stroke of a patient was not an objective in itself of our study but could be a direction for further research.

## 5. Conclusions

The supplementation of allopathic treatment with resveratrol had a beneficial effect on all monitored parameters that serve as major risk factors for stroke. The response to treatment was better in the 200 mg resveratrol group, but the differences were not significant compared to the 100 mg resveratrol group. Therefore, resveratrol can be used as a complementary, long-term therapy, as well as a beneficial adjuvant in the secondary prevention of stroke.

## Figures and Tables

**Table 1:** Demographic and clinical characteristics.

Characteristics	Resveratrol 100 mg group (*n* = 81)		Resveratrol 200 mg group (*n* = 55)		Control group (*n* = 92)	
	Number	%	Number	%	Number	%
*Gender*						
Females	32	39.51	21	38.18	37	40.22
Males	49	60.49	34	61.82	55	59.78
*Age*						
55–60 years	4	4.94	2	3.63	5	5.43
61–65 years	54	66.67	35	63.64	59	64.13
>65 years	23	28.39	18	32.73	28	30.44
Average	**65.03 ± 8.24**		**64.52 ± 8.05**		**64.78 ± 6.32**	
*Risk factors*						
Obesity	31	38.27	21	38.18	39	42.39
Toxicants (coffee, tobacco, and alcohol)	37	45.68	23	41.82	42	45.65
Stress	41	50.62	29	52.73	47	51.09
HBP	64	79.01	43	78.18	72	78.26
Diabetes	12	14.63	9	16.36	14	15.22
Dyslipidemia	50	61.73	37	67.3	61	66.30
*Diagnostic*						
Ischemic stroke	59	72.64	41	74.54	68	73.91
Hemorrhagic stroke	22	27.16	14	25.46	24	26.09
*Time since stroke occurred*						
<3 months	10	12.35	7	12.73	11	11.96
3–6 months	46	56.79	30	54.54	50	54.35
6–12 months	25	30.86	18	32.73	31	33.70
Average (months)	**5.08 ± 1.22**		**5.13 ± 1.14**		**5.17 ± 1.16**	

**Table 2:** Effect of resveratrol on blood pressure.

Evaluation	BP (mmHg)			
	Systolic BP	ES	Diastolic BP	ES
*Resveratrol 100 mg group*				
Initial	148.02 ± 15.28	—	88.29 ± 10.73	—
At 6 months	143.12 ± 15.16^∗^	0.32	85.89 ± 10.22	0.22
At 12 months	139.85 ± 14.82^∗c^	0.22	84.27 ± 10.18^∗^	0.16
*Resveratrol 200 mg group*				
Initial	149.21 ± 15.13	—	88.47 ± 11.11	—
At 6 months	142.02 ± 15.27^∗^	0.48	85.91 ± 11.02	0.23
At 12 months	139.35 ± 14.81^∗c^	0.17	84.10 ± 10.78^∗^	0.16
*Control group*				
Initial	148.42 ± 15.22	—	87.61 ± 11.25	—
At 6 months	146.10 ± 15.17	0.16	86.39 ± 11.32	0.11
At 12 months	145.32 ± 15.25	0.05	85.67 ± 11.11	0.06

Values are represented as mean ± SD (*n* = 81 patients in the resveratrol 100 mg group; *n* = 55 patients in the resveratrol 200 mg group; and *n* = 92 patients in the control group); ^∗^*p* < 0.05 versus initial value; ^c^<0.05 versus control group value.

**Table 3:** Effect of resveratrol on the weight status and on the body mass index.

Weight status	Resveratrol 100 mg group		Resveratrol 200 mg group		Control group	
	Number of patients	%	Number of patients	%	Number of patients	%
Normal weight	17	20.99	11	20.00	17	18.48
Overweight	33	40.74	23	41.82	36	39.13
Class I obesity	18	22.22	12	21.82	22	23.91
Class II obesity	9	11.11	7	12.73	12	13.04
Class III obesity	4	4.94	2	3.64	5	5.43
BMI average (initial)	**29.47 ± 4.26**		**29.50 ± 4.27**		**29.95 ± 4.49**	
Normal weight	21	25.93	15	27.27	17	18.48
Overweight	33	40.74	24	43.64	36	39.13
Class I obesity	16	19.75	9	16.36	23	25.00
Class II obesity	8	9.88	5	9.09	11	11.96
Class II obesity	3	3.70	2	3.64	5	5.43
BMI average (at six months)	**27.97 ± 4.44** ^c^		**27.56 ± 4.28** ^c^		**29.42 ± 4.72**	
ES	0.35		0.45		0.12	
Normal weight	28	34.57	19	34.55	18	19.57
Overweight	36	44.44	25	45.45	36	39.13
Class I obesity	10	12.35	6	10.91	22	23.91
Class II obesity	5	6.17	4	7.27	11	11.96
Class III obesity	2	2.47	1	1.82	5	5.43
BMI average (at 12 months)	**26.75 ± 4.12** ^∗c^		**25.77 ± 4.13** ^∗c^		**29.31 ± 4.55**	
ES	0.27		0.42		0.02	

Values are represented as mean ± SD (*n* = 81 patients in the resveratrol 100 mg group; *n* = 55 patients in the resveratrol 200 mg group; and *n* = 92 patients in the control group); ^∗^*p* < 0.05 versus initial value; ^c^<0.05 versus control group value.

**Table 4:** Effect of resveratrol on the lipid profile.

Lipid profile	Resveratrol 100 mg group		Resveratrol 200 mg group		Control group	
	M ± SD	ES	M ± SD	ES	M ± SD	ES
*Serum cholesterol (mg/dL)*						
Initial	257.56 ± 23.89	—	256.61 ± 22.23	—	255.14 ± 22.08	—
At 6 months	236.75 ± 22.82^∗c^	0.87	236.16 ± 22.61^∗c^	0.92	247.25 ± 21.67^∗^	0.36
At 12 months	221.52 ± 21.75^∗c^	0.67	220.44 ± 21.56^∗c^	0.70	231.41 ± 20.78^∗^	0.73
*HDL cholesterol (mg/dL)*						
Initial	39.27 ± 5.27	—	39.36 ± 5.16	—	38.76 ± 5.29	—
At 6 months	43.78 ± 5.28^∗c^	0.86	43.92 ± 5.18^∗c^	0.88	40.17 ± 5.21	0.27
At 12 months	45.87 ± 5.31^∗c^	0.40	46.12 ± 5.11^∗c^	0.42	42.02 ± 5.14^∗^	0.36
*LDL cholesterol (mg/dL)*						
Initial	143.34 ± 15.41		142.82 ± 15.26		143.48 ± 15.46	
At 6 months	127.66 ± 13.88^∗c^	1.02	125.01 ± 13.11^∗c^	1.04	137.92 ± 13.83^∗^	0.36
At 12 months	124.21 ± 13.67^∗c^	0.25	123.22 ± 13.13^∗c^	0.29	135.21 ± 13.60^∗^	0.20
*Triglycerides (mg/dL)*						
Initial	195.36 ± 20.16	—	195.69 ± 20.24	—	194.45 ± 20.24	—
At 6 months	172.73 ± 18.49^∗^	1.12	170.75 ± 18.23^∗c^	1.23	177.54 ± 19.01^∗^	0.84
At 12 months	158.78 ± 16.85^∗cr^	0.75	156.28 ± 16.28^∗c^	0.79	166.28 ± 17.77^∗^	0.59

Values are represented as mean ± SD (*n* = 81 patients in the resveratrol 100 mg group; *n* = 55 patients in the resveratrol 200 mg group; and *n* = 92 patients in the control group); ^∗^*p* < 0.05 versus initial value; ^c^<0.05 versus control group value; ^r^<0.05 versus resveratrol 200 mg group value.

**Table 5:** Effect of resveratrol on glucose.

Evaluation	Glucose (mg/dL)			
	Diabetics	ES	Nondiabetics	ES
*Resveratrol 100 mg group*				
Initial	142.18 ± 15.22	—	96.67 ± 11.26	—
At 6 months	136.29 ± 14.48^c^	0.39	91.23 ± 10.75^∗c^	0.48
At 12 months	134.24 ± 14.31	0.14	87.12 ± 10.61^∗c^	0.38
*Resveratrol 200 mg group*				
Initial	142.46 ± 15.24	—	96.98 ± 11.23	—
At 6 months	136.21 ± 14.56^c^	0.41	91.21 ± 10.67^∗c^	0.51
At 12 months	133.89 ± 15.68	0.16	86.11 ± 10.78^∗c^	0.48
*Control group*				
Initial	142.47 ± 15.83	—	95.71 ± 11.63	—
At 6 months	141.81 ± 15.31	0.04	94.55 ± 11.22	0.10
At 12 months	140.58 ± 15.62	0.08	93.33 ± 11.28	0.11

Values are represented as mean ± SD (*n* = 13 diabetic and *n* = 68 nondiabetic patients in the resveratrol 100 mg group; *n* = 11 diabetic and *n* = 44 nondiabetic patients in the resveratrol 200 mg group; and *n* = 16 diabetic and *n* = 76 nondiabetic patients in the control group); ^∗^*p* < 0.05 versus initial value; ^c^<0.05 versus control group value.

**Table 6:** Effect of resveratrol on HbA1c.

Evaluation	HbA1c (%)					
	Resveratrol 100 mg group		Resveratrol 200 mg group		Control group	
	M ± SD	ES	M ± SD	ES	M ± SD	ES
Initial	7.13 ± 1.51	—	7.15 ± 1.50	—	7.10 ± 1.47	—
At 6 months	6.72 ± 1.40	0.27	6.66 ± 1.41	0.33	7.09 ± 1.43	0.01
At 12 months	6.61 ± 1.38	0.08	6.55 ± 1.40	0.08	7.05 ± 1.46	0.03

Values are represented as mean ± SD (*n* = 13 diabetic patients in the resveratrol 100 mg group; *n* = 11 diabetic patients in the resveratrol 200 mg group; and *n* = 16 diabetic patients in the control group).

## Abbreviations

BMI: Body mass index
BP: Blood pressure
DNA: Deoxyribonucleic acid
ES: Effect size
HbA1c: Glycosylated hemoglobin
HDL: High-density lipoprotein
LDL: Low-density lipoprotein
ROS: Reactive oxygen species
SIRT1: Sirtuin 1
AMPK: Adenosine monophosphate-activated kinase.
